# The association between SGLT2 inhibitors and new-onset acute coronary syndrome in the elderly: a population-based longitudinal cohort study

**DOI:** 10.1186/s13098-023-01143-5

**Published:** 2023-08-17

**Authors:** Tsung-Kun Lin, Mei-Chun Lee, Yu-Han Cheng, Timothy Ma, Mei-Chun Chen, Tsung-Yuan Yang, Gwo-Ping Jong

**Affiliations:** 1 Department of Pharmacy, Taoyuan Armed Forces General Hospital, Taoyuan, Taiwan, ROC; 2 School of Pharmacy, National Defense Medical Center, Taipei, Taiwan, ROC; 3 Department of Pharmacy, Mackay Memorial Hospital, Taipei, Taiwan, ROC; 4 Nursing and Management, Mackay Junior College of Medicine, Taipei, Taiwan, ROC; 5 Department of Internal Medicine, Tri-Service General Hospital, Taipei, Taiwan, ROC; 6 Department of Medical Supply, Carle Hospital, Urbana, IL 61801 USA; 7 Division of Cardiology, Department of Internal Medicine, Chung Shan Medical University Hospital, Taichung, Taiwan, ROC; 8 Institute of Medicine, Chung Shan Medical University, Taichung, Taiwan, ROC

**Keywords:** Sodium–glucose co-transporter inhibitors, Diabetes mellitus, Acute coronary syndrome

## Abstract

**Background:**

Several observational cohorts and meta-analytical studies on humans have shown that users of sodium-glucose cotransporter-2 inhibitors (SGLT2is) have a lower risk for new-onset acute coronary syndrome (ACS) than nonusers. However, some studies, including randomized clinical trials, reported the opposite results. This study aimed to investigate the impacts of a SGLT2i on new-onset ACS in a population.

**Methods:**

We conducted a retrospective population-based cohort study involving 56,356 subjects who received SGLT2i therapy and 112,712 patients who did not receive SGLT2i therapy between May 1, 2016 and December 31, 2019. The outcome was the risk of new-onset ACS. Multivariable Cox proportional hazards models were used to calculate hazard ratios (HRs) and 95% confidence intervals for associations between SGLT2i use and ACS risk.

**Results:**

A total of 670 and 1408 ACS events occurred in SGLT2i users and nonusers, respectively, during a follow-up of 3.7 years. SGLT2i use was associated with a nonsignificantly lower risk of ACS (adjusted HR 0.95, 95%confidence intervals (CI 0.87–1.04, P = 0.3218). We confirmed the robustness of these results through a propensity score 1:1 matching analysis. The results of the subgroup analysis of the subtype of the SGLT2i treatments were consistent with the main findings. An increased risk for the incidence of ACS in male and older (> 70 years) patients were also found.

**Conclusions:**

In this population-based cohort study, we found that SGLT2i use is associated with a nonsignificantly decreased risk of ACS. No difference in the SGLT2i subtype was observed in subgroup analyses. However, the results of this study indicated an increased risk for the incidence of ACS in male and older (> 70 years) patients.

## Introduction

Type 2 diabetes (T2D) and acute coronary syndrome (ACS) are associated with significant morbidity and mortality despite advances in their clinical management [1, 2]. The prevalence and incidence of T2D and ACS remains high and have imposed considerable health burden worldwide [3]. Patients with T2D and ACS are prone to developing sudden cardiac death, which contributes to carbonyl stress, polyol pathway, oxidative stress, hexosamine pathways, diacylglycerol/protein kinase-C activation, and structural remodeling as well as insulin resistance and glycemic fluctuations [4]. The primary and secondary prevention of ACS in patients with T2D through aggressive pharmacologic and nonpharmocologic management has been proposed as the most effective way to reduce the incidence, severity, and long-term complications of ACS [5, 6].

When used as an antidiabetic drug, sodium-glucose cotransporter-2 inhibitors (SGLT2is) have been proven to have a cardiovascular protective effect in reducing major cardiovascular events and heart failure and to have greater benefits in patients with established atherosclerotic cardiovascular diseases [7, 8]. Clinical trials have also shown that SGLT2is reduced cardiovascular death or urgent heart failure visit regardless of diabetes status [9, 10]. However, the association between SGLT2i use and ACS risk in patients with T2D remains inconsistent in real-world practice [11–13]. The purpose of the present study is to evaluate the risk for ACS associated with the prescription of SGLT2is in a population-based cohort study of T2D in Taiwan.

## Methods

### Study design and population

In this retrospective cohort study, patient data were obtained from the National Health Insurance (NHI) program, which is a compulsory universal health insurance program in Taiwan and covers approximately 99% of Taiwanese residents [14]. The NHI database stores information, including claim forms, and contains patient sex; age; three diagnostic codes; medical expenditures; and prescriptions, such as drug quantity and expenditure, drug dose, operations, and treatments. All personal information was encrypted and deidentified to preserve patient privacy.

This study was approved by the Ethics Committee of the Tri-Service General Hospital (B-202205007). Written consent was not obtained from the study participants because only deidentified data were obtained from the Longitudinal Health Insurance Database, and a waiver of patient consent was provided by the Ethics Committee for this study.

### Data collection and definitions

This study extracted data from the NHI program in Taiwan from January 2010 to December 2019 by using newly diagnosed type 2 DM codes based on the International Classification of Diseases (ICD), ninth revision, Clinical Modification (CM) (ICD-9-CM) and ICD, tenth revision, CM (ICD-10-CM). Newly diagnosed T2D was defined as the first time that a T2D code was available in outpatient or inpatient claim records between January 2010 and December 2019.

This study included adults (aged ≥ 65 years) with T2D ICD-9-CM code 250 or ICD-10-CM code E11 who were treated with the maximum tolerated labeled dose of a SGLT2i for more than 180 days. The patients were identified as inpatients or outpatients between May 2016 and December 2019.

**Fig. 1:**
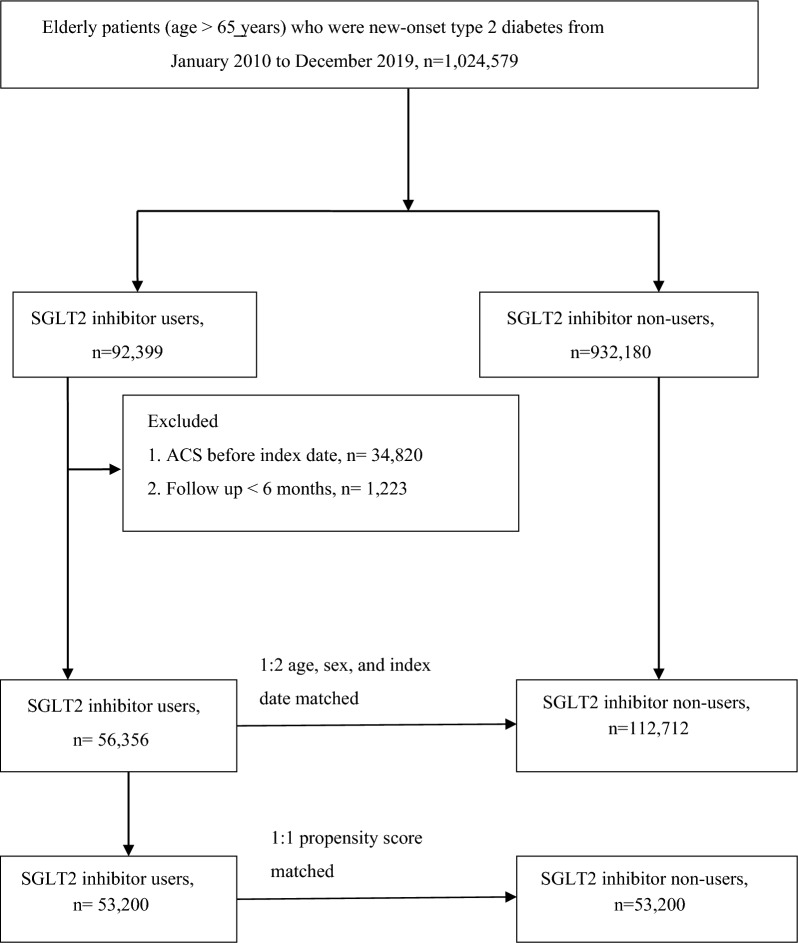
Flowchart of study population

The participants had to meet at least one of the following criteria: (1) had two or more outpatient visits within 6 months, (2) continuously received antidiabetic medication for more than 6 months during the study period, or (3) had one or more inpatient admissions with a diagnosis of T2D. Comorbidities related to ACS were recorded in accordance with the ICD-10-CM code and included coronary heart disease (ICD-10-CM code I20–I25), hypertension (ICD-10-CM code I10), hyperlipidemia (ICD-9-CM code E78.1–E78.5), chronic liver disease (ICD-10-CM codes K71, K75, and K76), chronic obstructive pulmonary disease (ICD-10-CM code J44), atrial fibrillation and flutter (ICD-10-CM code I48), and rheumatoid arthritis (ICD-9-CM code M05). Exclusion criteria included (1) a prior history of ACS before May 2016, (2) follow-up period of less than 6 months, and (3) age less than 65 years. The SGLT2i and non-SGLT2i groups were matched for age, sex, and T2D duration at a ratio of 1:2. The final study sample comprised 56,356 SGLT2i users and 112,712 non-SGLT2i users (Fig. 1). Sensitivity analysis using propensity score matching was also performed with a matching ratio of 1:1 (Fig. 1).

### Variables and outcomes

Patient demographic characteristics were assessed on the index date. Demographic variables included gender, age, diabetes duration, comorbidities, concurrent medication, and SGLT2i type. Comorbidities closest to the index date within 7 days and medication use were assessed during a 180-day baseline period. The study endpoint was new-onset ACS, defined as the first occurrence of an ACS code (ICD-10-CM codes I60, I61, I62, I63, I65, I66, I67.84, G45, and G46) in inpatient or outpatient claim records during follow-up.

### Statistical analyses

Baseline demographic/clinical characteristics were compared between the two study groups. Student’s t-test and chi-square test were used to evaluate the distribution of continuous and categorical variables for a matched cohort. Standardized mean difference ≤ 0.10 indicates a negligible difference in potential confounders between the two cohorts. In both cohorts, the incidence rates of suicide were calculated as per 10,000 person-months. The crude hazard ratios (HRs) and 95% confidence intervals (CIs) of new-onset ACS were estimated by using Cox proportional hazard regression. Multivariable models were further adjusted for important risk factors for developing ACS, including comorbidities and concurrent medication. The risk of ACS over time for the SGLT2i group compared with that for the non-SGLT2i group was determined through survival analysis with the Kaplan–Meier method. Because of the observed differences in baseline characteristics existed, the propensity score matching was performed with a matching ratio of 1:1 to balance baseline covariates between two groups for sensitivity analysis. Subgroup analyses stratified by SGLT2i type was performed on the outcomes. Statistical significance was defined at *P*-value < 0.05. All statistical analyses were performed by using SAS 9.4 (SAS Institute Inc., Cary, North Carolina, USA).

## Results

### Baseline characteristics

After 1:2 age, sex, and index date matching, we found 56,356 patients on a SGLT2i and 112,712 patients not on a SGLT2i. The baseline characteristics of all patients in the SGLT2i and non-SGLT2i groups are presented in Table 1. In each group, the majority of patients had a T2D duration of 3–4 years (59.76% versus 61.19%). In both groups, most subjects were females (53.51%) and 65–69 years of age (48.00%). In both groups, the mean age was 71.4 ± 5.8 years. Compared with non-SGLT2i users, SGLT2i users had a higher proportion of comorbidities with hypertension, hyperlipidemia, chronic kidney disease, and atrial fibrillation and flutter. SGLT2i users had lower rates of comorbidities with chronic liver disease, cancer, chronic obstructive pulmonary disease, rheumatoid arthritis, and stroke than non-SGLT2i users. The proportion of patients receiving a corticosteroid, aspirin, statin, biguanide, sulfonylurea, alpha glucosidase inhibitor, thiazolidinedione, insulin, and glucagon-like peptide-1 agonist was higher in those under SGLT2i therapy than in those not under SGLT2i therapy. The proportion of prescription with a nonsteroidal anti-inflammatory drug, H2 blocker, and dipeptidyl peptidase-4 inhibitor was lower in patients under SGLT2i treatment that in those not under SGLT2i treatment.

**Table 1:** Baseline characteristics of all patients

	2:1 sex, age, and index date matching				PS matching			
	Non-SGLT2i	SGLT2i	ASD	*P* value	Non-SGLT2i	SGLT2i	ASD	*P* value
N	112712	56356			53200	53200		
Type 2 DM history			0.0222	< 0.0001			0.0387	0.0036
< = 2 years	10527 (9.34%)	4885 (8.67%)			4406 (8.28%)	4701 (8.84%)		
3–4 years	68969 (61.19%)	33677 (59.76%)			31845 (59.86%)	31784 (59.74%)		
> = 5 years	33216 (29.47%)	17794 (31.57%)			16949 (31.86%)	16715 (31.42%)		
Sex			0.0000	1.000			0.0122	0.0463
Female	60308 (53.51%)	30154 (53.51%)			28836 (54.20%)	28512 (53.59%)		
Male	52404 (46.49%)	26202 (46.49%)			24364 (45.80%)	24688 (46.41%)		
Age			0.0000	1.000			0.0346	0.5766
65–69	54106 (48.00%)	27053 (48.00%)			25584 (48.09%)	25559 (48.04%)		
70–79	46784 (41.51%)	23392 (41.51%)			21976 (41.31%)	22094 (41.53%)		
80 up	11822 (10.49%)	5911 (10.49%)			5640 (10.60%)	5547 (10.43%)		
Mean ± SD	71.43 ± 5.75	71.43 ± 5.75			71.42 ± 5.72	71.43 ± 5.75		
Comorbidities								
Hypertension	71523 (63.46%)	37189 (65.99%)	0.0530	< 0.0001	35631 (66.98%)	34986 (65.76%)	0.0257	< 0.0001
Hyperlipidemia	59503 (52.79%)	33545 (59.52%)	0.1360	< 0.0001	31906 (59.97%)	31311 (58.86%)	0.0228	0.0002
Chronic kidney disease	31434 (27.89%)	16886 (29.96%)	0.0458	< 0.0001	16039 (30.15%)	15781 (29.66%)	0.0106	0.0841
Chronic liver disease	9463 (8.40%)	4647 (8.25%)	0.0054	0.2933	4308 (8.10%)	4388 (8.25%)	0.0055	0.3707
Cancer	8655 (7.68%)	3651 (6.48%)	0.0468	< 0.0001	3357 (6.31%)	3523 (6.62%)	0.0127	0.0385
COPD	5694 (5.05%)	2466 (4.38%)	0.0319	< 0.0001	2254 (4.24%)	2364 (4.44%)	0.0102	0.0979
Atrial fibrillation and flutter	1976 (1.75%)	1248 (2.21%)	0.0331	< 0.0001	1148 (2.16%)	1147 (2.16%)	0.0001	0.9832
Rheumatoid Arthritis	1012 (0.90%)	382 (0.68%)	0.0249	< 0.0001	335 (0.63%)	371 (0.70%)	0.0083	0.1740
Stroke	8864 (7.86%)	4068 (7.22%)	0.0245	< 0.0001	3736 (7.02%)	3923 (7.37%)	0.0136	0.0266
Medication								
NSAIDs	63911 (56.70%)	31616 (56.10%)	0.0122	0.0185	29889 (56.18%)	29885 (56.17%)	0.0002	0.9803
Corticosteroids	20924 (18.56%)	10060 (17.85%)	0.0185	0.0004	9334 (17.55%)	9550 (17.95%)	0.0106	0.0831
PPI	7949 (7.05%)	3890 (6.90%)	0.0059	0.2548	3462 (6.51%)	3661 (6.88%)	0.0150	0.0147
H2 receptor antagonist	31112 (27.60%)	14764 (26.20%)	0.0317	< 0.0001	13890 (26.11%)	14068 (26.44%)	0.0076	0.2150
Aspirin	29056 (25.78%)	17682 (31.38%)	0.1241	< 0.0001	16292 (30.62%)	16217 (30.48%)	0.0031	0.6177
Statin	62219 (55.20%)	39702 (70.45%)	0.3195	< 0.0001	37187 (69.90%)	36688 (68.96%)	0.0204	0.0009
Biguanides	56490 (50.12%)	31500 (55.89%)	0.1159	< 0.0001	28410 (53.40%)	29253 (54.99%)	0.0318	< .0001
Sulfonylureas	40100 (35.58%)	22165 (39.33%)	0.0776	< 0.0001	21082 (39.63%)	20618 (38.76%)	0.0179	0.0036
Alpha glucosidase inhibitors	12020 (10.66%)	10003 (17.75%)	0.2040	< 0.0001	8564 (16.10%)	8478 (15.94%)	0.0044	0.4722
Thiazolidinediones	11,219 (9.95%)	9053 (16.06%)	0.1824	< 0.0001	7887 (14.83%)	7943 (14.93%)	0.0030	0.6295
DPP4	27889 (24.74%)	8422 (14.94%)	0.2476	< 0.0001	8650 (16.26%)	8354 (15.70%)	0.0152	0.0137
Insulin	14052 (12.47%)	11579 (20.55%)	0.2189	< 0.0001	9561 (17.97%)	9797 (18.42%)	0.0115	0.0607
GLP-1	621 (0.55%)	746 (1.32%)	0.0803	< 0.0001	538 (1.01%)	580 (1.09%)	0.0077	0.2067
SGLT2i subtype			–				–	
Dapagliflozin	0 (0%)	30225 (53.63%)			0 (0%)	28604 (53.77%)		
Canagliflozin	0 (0%)	1056 (1.87%)			0 (0%)	1006 (1.89%)		
Empagliflozin	0 (0%)	25075 (44.49%)			0 (0%)	23590 (44.34%)		

*SGLT2i* Sodium-glucose cotransporter-2 inhibitor, *COPD* chronic obstructive pulmonary disease, *DM* Diabetes Mellitus, *GLP-1* Glucagon-like peptide-1, *DPP4* Dipeptidyl peptidase-4, *NSAID* Non-steroidal anti-inflammatory drug, *PPI* Proton-pump inhibitor, *ASD*: absolute standardized difference, *PS* propensity score, *PS* propensity score

### ACS during follow-up

**Fig. 2:**
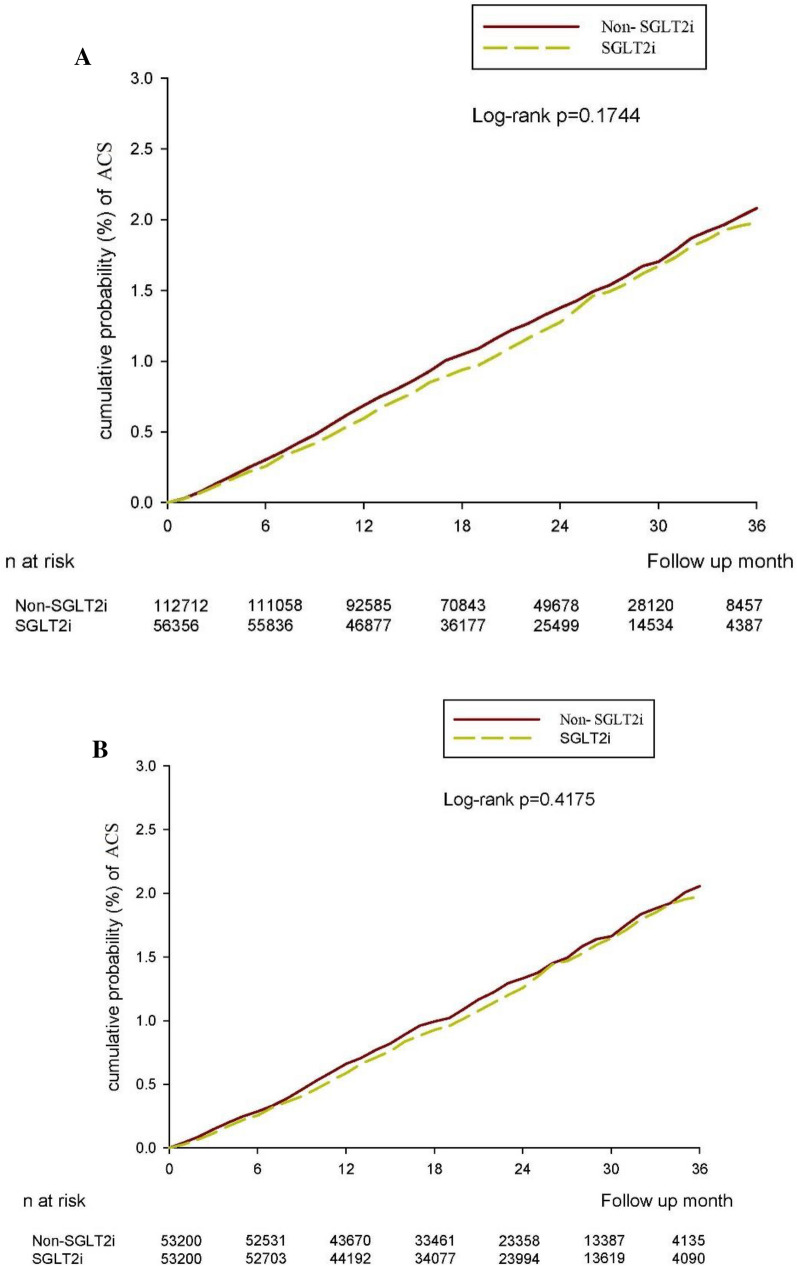
**A** Cumulative risk curve of new-onset ACS for the study cohorts treated with SGLT2 inhibitors versus non-SGLT2 inhibitors user. **B** Cumulative risk curve of new-onset ACS for the study cohorts under propensity score matching treated with SGLT2 inhibitors versus non-SGLT2 inhibitors users

The follow-up periods were 1,180,118 person-months in the SGLT2i group and 2,325,847 person-months in the non-SGLT2i group. The patients with SGLT2i treatment had lower incidence rates of ACS than the patients not receiving SGLT2i treatment (5.68 versus 6.05 per 10,000 person-months). The SGLT2i group showed a nonsignificant association with a lower risk of ACS (HR 0.94, 95% CI 0.86–1.03, *P* = 0.1763) (Table 2). The result obtained after multiple Cox proportional hazards model analysis was consistent with the above finding (HR 0.95, 95% CI 0.87–1.04, *P* = 0.3218). Kaplan–Meier analysis also demonstrated that the cumulative probability of ACS was nonsignificantly lower in patients with SGLT2i (*P* = 0.1744) (Fig. 2A).

**Table 2:** Incidence rate of acute coronary syndrome in study groups

	2: 1 sex, age, and index date matching		*P* value	PS matching		*P* value
	Non- SGLT2i	SGLT2i		Non- SGLT2i	SGLT2i	
N	112712	56356		53200	53200	
Follow up person months	2325847	1180118		1099285	1111867	
New case	1408	670		646	624	
Incidence rate^a^(95% C.I.)	6.05(5.75–6.38)	5.68(5.26–6.12)		5.88(5.44–6.35)	5.61(5.19–6.07)	
Crude Relative risk (95% C.I.)	Reference	0.94(0.86–1.03)	0.1763	Reference	0.96(0.86–1.07)	0.4110
Adjusted HR^a^ (95% C.I.)^b^	Reference	0.95(0.87–1.05)	0.3218	Reference	0.96(0.86–1.07)	0.4111

*SGLT2i* Sodium-glucose cotransporter-2 inhibitor

^a^Incidence rate, per 10,000 person-months

^b^ adjusted hazard ratio, the covariates including year of index, sex, age, co-morbidities, and medication at baseline

PS: propensity score

### Sensitivity and subgroup analysis

We performed sensitivity analysis on participants with a propensity score 1:1 matching analysis. The association between SGLT2i use and ACS incidence did not change when compared with the main results (aHR: 0.96, 95% CI 0.86–1.07, Table 2; or Fig. 2B). The subtype of SGLT2i was analyzed to compare the HRs (95% CIs) of study outcomes between the SGLT2i group and the non-SGLT2i group (Table 3). The result was also consistent with the findings of the main analyses (dapagliflozin aHR: 0.99, 95% CI 0.89–1.12; canagliflozin aHR: 0.58, 95% CI 0.19–1.82; empagliflozin aHR: 0.91, 95% CI 0.80–1.03 in Table 3). However, among SGLT2i users, males were at significantly higher risk for ACS than females (aHR: 1.47; 95% CI 1.34–1.60). Moreover, female patients in the SGLT2i user group had a significant lower risk of new-onset ACS compared to those in the SGLT2i nonuser group (aHR: 0.88, 95% CI 0.78–1.00; *P* = 0.0495). Furthermore, compared with younger patients (aged 65–69), elderly patients (aged > 70) exhibited a significantly higher risk for ACS (70–79 vs. 65–69 years, aHR: 1.33, 95% CI 1.21–1.47; > 80 vs. 65–69 years, aHR: 1.89, 95% CI 1.66–2.15 in Table 3).

**Table 3:** Multiple variables Cox regression to estimate the hazard ratio (HR) in subgroup analysis

	aHR(95% CI)^a^	
	2:1 sex, age, and index date matching	PS matching
Study		
Non-SGLT2i	Reference	Reference
SGLT2i	0.95(0.87–1.05)	0.96(0.86–1.07)
SGLT2i subtype(ref: non)		
Dapagliflozin	0.99(0.89–1.12)	0.99(0.87–1.13)
Canagliflozin	0.58(0.19–1.82)	0.63(0.20–1.97)
Empagliflozin	0.91(0.80–1.03)	0.91(0.79–1.05)
Type 2 DM history		
< = 2 years	1.46(1.23–1.73)	1.53(1.24–1.9)
3–4 years	1.17(1.04–1.32)	1.16(0.99–1.35)
> = 5 years	Reference	Reference
Sex		
Female	Reference	Reference
Male	1.47(1.34–1.60)	1.41(1.26–1.58)
Female (users vs. non-users)	0.88(0.78–1.00)	0.90(0.79–1.02)
Male (users vs. non-users)	0.99(0.87–1.13)	0.98(0.86–1.11)
Age		
65–69	Reference	Reference
70–79	1.33(1.21–1.47)	1.34(1.19–1.51)
80 up	1.89(1.66–2.15)	2.00(1.70–2.36)

*SGLT2i* Sodium-glucose cotransporter-2 inhibitor, *DM* Diabetes Mellitus, *PS* propensity score

^a^ adjusted hazard ratio, the covariates including year of index, sex, age, co-morbidities, and medication at baseline

## Discussion

In this population-based cohort study, we observed that patients taking SGLT2i had a nonsignificant lower risk for ACS than non-SGLT2i users. Sensitivity and Kaplan–Meier analyses also demonstrated a nonsignificantly lower risk for ACS in the patients in the SGLT2i group. No difference in SGLT2i subtype was observed in subgroup analyses. However, the results of this study indicated an increased risk for the incidence of ACS in male and older (> 70 years) patients.

SGLT2is are approved for lowering glucose in patients with T2D and reducing cardiovascular events [15]. The mechanism of action of SGLT2i is to promote glucose excretion in urine by blocking glucose and sodium reuptake in the early proximal renal tubule and thereby increasing glycosuria and natriuresis [16, 17]. In a randomized control trial on patients with T2D, Kosiborod et al. compared SGLT2i and other glucose-lowering drugs and found a significant reduction in hospitalization due to heart failure, the incidence of myocardial infarction, and all-cause mortality [18]. Another randomized control trial by Zinman et al. discovered that using the drug empagliflozin lowered the risk of death from cardiovascular causes, nonfatal MI, or nonfatal stroke in comparison with the use of a placebo [7]. However, no randomized control trial showing that SGLT2i could significantly reduce the risk for the incidence of ACS in patients with T2D has been conducted. In the present cohort study, we observed that taking SGLT2i had a potential protective effect against new-onset ACS. Till now, little data are available regarding therapeutic strategies to reduce the incidence of ACS in these patients with T2D and use SGLT2i. The mechanisms involved in SGLT2i administration and ACS remain unknown.

Our study found a gender difference in the incidence of ACS in patients with T2D between the SGLT2i and non-SGLT2i users in our study. Similarly, previous studies have demonstrated gender differences in SGLT2i treatment across a variety of study populations [19–21]. Wang et al. evaluated 11,007 patients with type 2 DM, of whom 3856 (35%) were women. After adjusting for baseline differences, they found that women were less likely than men to experience total cardiovascular events (aHR 0.77, 95% CI 0.71–0.84) [21]. However, Rådholm et al. enrolled patients from the EMPA-REG OUTCOME, CANVAS Program, DECLARE TIMI-58, and CREDENCE trials and found no gender differences in the risk for major adverse cardiovascular events between SGLT2i users and nonusers [22]. T2D confers a differential risk for cardiovascular disease according to gender. To date, no consensus has been reached regarding the gender-related difference in the incidence of ACS between patients with T2D with or without SGLT2i use. Further randomized trials are needed to explore the gender-related difference between SGLT2i prescription and ACS incidence.

In most other clinical trials, old age was clearly a risk factor for developing ACS [23]. In the present study, SGLT2i use resulted in a significantly deleterious effect against the incidence of ACS in users aged > 70 years relative to in users aged 65–70 years. The age-related differences in SGLT2i use can be attributed to differences in longevity, survival bias, and comorbidities (e.g., DM, hypertension, and chronic obstructive lung disease) between older and young patients [24, 25]. Another reason is that the elderly are less likely to report having been offered SGLT2i therapy and more likely to decline compliance when offered [26]. Further comprehensive clinical research is warranted to elucidate the mechanisms underlying this association.

Consistent with the results reported in previous literature, our findings showed no significant difference in the risk of developing ACS among patients taking dapagliflozin, canagliflozin, and empagliflozin [11, 27]. Suzuki et al. evaluated 25,315 patients and found that the risks for the subsequent development of MI were comparable among dapagliflozin, canagliflozin, and empagliflozin [27]. However, an analysis of a retrospective cohort showed that dapagliflozin might have a more favorable effect on cardiovascular outcomes than empagliflozin [28]. Another study also reported that canagliflozin and empagliflozin were most effective for the prevention of heart failure hospitalization than other SGLT2is [29]. Therefore, whether the risk of ACS differs among individual SGLT2i is uncertain.

### Strengthens and limitations

The strengthens of our study included a population-based nature large sample size, and in real-world data. However, our study also has are several limitations. First, the retrospective data-based secondary analysis.

has certain inherent limitations in this study. Second, the laboratory data such as fasting blood sugar levels, hemoglobin A1c levels, liver function, and some imaging findings important factors related to ACS prevention such as smoking, BMI, family history, diet habits and data were not available from these NHI data. However, because the data we used were population-based data, we assumed that there were no differences among the two groups. Third, other residual confounding factors, such as genetics, physical activity or dietary factors, are also not included in the NHI data. However, our results are consistent with those of previous validation studies [19, 20]. Fourth, Three types of SGLT2i (Empagliflozin, Dapagliflozin, and Canagliflozin) were launched since May 2016 and used till the end of the study (December 31, 2019). Follow-up is still relatively short, and it is possible that effects of SGLT2i user may take much longer than 3.7 years to become statistical significance.

## Conclusion

Patients with T2D taking SGLT2is are associated with a nonsignificantly decreased risk of ACS compared with those without SGLT2is prescription in real-world practice. No difference in SGLT2is subtype was also observed in this study. However, a gender-related difference in the incidence of ACS between patients with T2D with or without SGLT2i use. Further comprehensive clinical research may be needed to explore the gender-related difference between SGLT2i prescription and ACS incidence.

## Data Availability

All the re-identified data are available upon reasonable request (cgp8009@yahoo.com.tw).

## Abbreviations

T2D: Type 2 diabetes
ACS: Acute coronary syndrome
SGLT2i: Sodium-glucose co-transporter inhibitor
NHI: National Health Insurance
ICD-9-CM: International Classification of Diseases, ninth revision; Clinical Modification
ICD-10-CM: International Classification of Diseases, tenth revision, Clinical Modification
HR: Hazard ratio
CI: Confidence interval
